# Artificial intelligence catalyzes antimicrobial peptide design

**DOI:** 10.1016/j.synbio.2026.05.019

**Published:** 2026-07-28

**Authors:** Yongqiang Liu, Jie Hu, Ning Zhang, Yinqi Bai, Yuan Yao, Gaoxiang Chen

**Affiliations:** a Zhejiang Lab, Hangzhou, 311121, Zhejiang, China; b Medical Artificial Intelligence Center, Hangzhou Institute of Medicine, Chinese Academy of Sciences, Hangzhou, 310000, Zhejiang, China; c BGI Research, Hangzhou, 310030, Zhejiang, China; d BGI Research, Sanya, 572025, Hainan, China; e Zhejiang Key Laboratory of Intelligent Manufacturing for Functional Chemicals, ZJU-Hangzhou Global Scientific and Technological Innovation Center, Zhejiang University, Hangzhou, 311215, Zhejiang, China; f Zhejiang Synthetic Biomanufacturing Innovation Center, Hangzhou, 311215, Zhejiang, China; g Department of Food Science and Technology, School of Agriculture and Biology, Shanghai Jiao Tong University, Shanghai, 200240, Zhejiang, China

**Keywords:** Antimicrobial peptides, Antimicrobial resistance, Microorganism, Computational biology, Artificial intelligence

## Abstract

With broad-spectrum, low resistance, and multifunctional properties, antimicrobial peptides (AMPs) are promising therapeutic agents against drug-resistant pathogens, yet their discovery and optimization still remain challenging due to the complexity of sequence-function associations. Artificial intelligence (AI), through the construction of comprehensive data-driven models that assisted with miscellaneous learning strategies, enables de novo peptide design by learning latent representations inherent in peptide sequences as well as their biological properties to ensure physically plausible and biologically relevant predictions. Consequently, this paradigm enhances the likelihood of designing peptide candidates with significantly improved therapeutic potential, reducing resource-intensive trial-and-error processes and revealing the transformative impact of computational innovation in advancing next-generation therapeutics. Here, we provide a snapshot of this field and survey two modes of AI-driven technologies for AMP design, one concentrated on identifying whether current data possess antimicrobial activity (identification-oriented) and the other on generating AMP candidates with potential therapeutic properties (generation-oriented). We also highlight the challenges and limitations that still hinder AMP development even accelerated by AI, as well as the foreseeable prospects, from finer-grained explorations to model-driven data enrichment and model enhancement.

## 1 Introduction

Antibiotics [1], [2], [3], [4], [5], [6], [7] exerted a significant influence on mitigating global mortality incurred by microbial infections. However, the emergence of antimicrobial resistance and other accompanying long-lasting side-effects severely limited their advancement in the pharmaceutical field [8], [9], [10], [11], [12], [13], [14], [15], [16], [17]. Antimicrobial peptides (AMPs) [18], [19], [20], [21], [22], [23], [24], [25], a part of the immune defense family that always consists of short amino acid sequences, possess the characteristics that enable the termination of various bacteria via mechanisms such as disrupting cell membranes, immunomodulation, specific target binding, and interference with metabolic processes [26], [27], [28], [29]. Consequently, AMPs have emerged as effective candidates for the development of novel alternative therapies. Their broad-spectrum activity and lower risk of antimicrobial resistance position them as super-potential agents in drug development, thereby mitigating the threat posed to the lives of patients and the economy of our society [30], [31]. The extensive exploration of AMPs from diverse sources, encompassing both natural and synthetic origins, has demonstrated their capacity to combat microbial pathogens. In addition to their antimicrobial properties, AMPs have also been identified as conferring additional benefits, such as promoting wound healing, exhibiting anti-tumor activities, and modulating immune responses [32], [33], [34], [35]. Recombinant or synthetic AMPs offer a viable and safe alternative for therapeutic applications in aquaculture [36], [37], [38], plant protection [39], food preservatives [40], [41], [42], [43], and even in biomaterials [44], [45], [46], [47], [48]. The heightened imperative to identify and design more effective AMPs has thus been underscored.

However, the systematic exploration and stepwise refinement of AMPs for clinical translation is a complex, multidisciplinary endeavor. It demands integration across diverse fields such as biology, materials science, chemistry, statistics, computer science, bioinformatics, and molecular informatics, posing significant challenges. Moreover, recent evidence reveals that AMPs exhibit an unexpected level of specificity and significant potential for synergistic interactions [49], [50], [51], [52], [53], [54]. This indicates that studying a single AMP in isolation may be insufficient to fully comprehend its mechanism of action. Instead, combining it with other antimicrobial peptides or antibiotics could potentially achieve a more potent therapeutic effect, which adds to the complexity of exploring and understanding the mechanisms. To this end, close cooperation among multiple parties is regarded as the key to success. Meanwhile, the employment of an intelligent strategy or a versatile tool [55] can certainly better facilitate and accelerate the process of exploring AMPs.

**Fig. 1:**
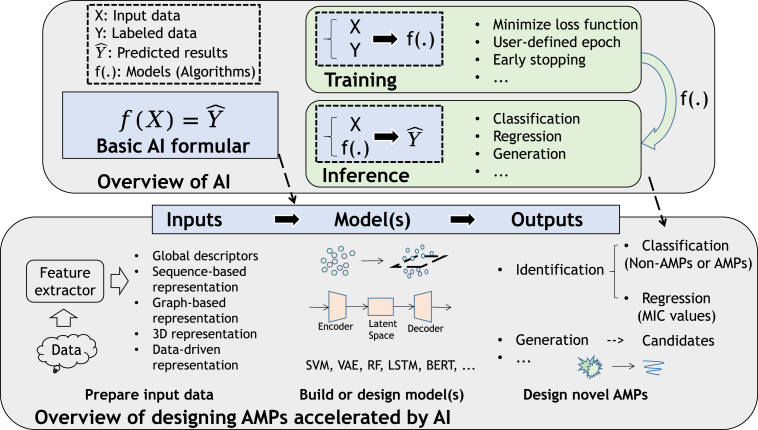
Overview of designing AMPs accelerated by AI.

As a transformative technology accelerating progress across multiple domains, artificial intelligence (AI) [56], [57], [58], [59], [60], [61], [62], [63], [64] has emerged as the brightest rising star, capturing widespread attention. Individual intelligence systems, along with associated methods and models, have been widely and successfully applied in multiple fields such as natural language processing, image recognition, robotics, and games. By training complex weight-driven models (such as deep neural networks, DNNs) to learn patterns and representations from large amounts of data, these methods are able to generate new required data or make decisions or predictions for various tasks. In the past few years, to get rid of the limitations that time-consuming and low-throughput brings in biological exploration, these AI-based learning models have made a lot of contributions, e.g., genomic analysis [65], [66], [67], [68], structural modeling and prediction [69], [70], [71], [72], [73], [74], medical imaging and disease diagnosis [75], [76], and novel drug discovery [77], [78], [79], [80], [81].

To date, numerous computational methodologies have been devised for expediting the design of novel AMPs. In this review, we survey how these AI-based models have assisted various aspects of AMPs’ design. Our analytical synthesis traverses the developmental arc of AI-assisted exploration of AMPs. We present a snapshot of the operational intricacies inherent to these methodologies, delineating the sequential stages of data aggregation, dataset feature extraction, model training, and inference. Particularly, we investigate two crucial modes for AMP design, the identification-oriented mode and the generation-oriented mode, as well as an overview of the methodologies employed therein. Finally, we analyze the prevailing challenges impeding AMP advancement and proffer prospective developments in this field.

## 2 Preliminaries

With lower risk of antimicrobial resistance, broad-spectrum antibacterial activity, low propensity to develop toxicity, multiple anti-activity (antibacterial, antiviral, antifungal, anticancer, antiprotozoal, etc.) [82], [83], [84], [85], [86], AMP has emerged as a treasure aiding clinical therapeutics and a warrior fighting against kinds of diseases, such as respiratory diseases [87], [88], [89], [90], eyes diseases [91], [92], gastrointestinal diseases [93], [94], [95], [96], bone and joint infection [97], [98], wound healing and skin infections [99], [100], and oral diseases [101], [102]. Typically, they rely on disrupting cell membranes to destroy targets [103], [104], interfere with metabolic processes, or promote immune cell activation and immune response by lowering the barrier to cell entry [105], [106], [107]. During the detailed exploration of AMPs’ mechanisms of action, some external manifestation rules have been summarized, as follows.

• **Element-level**. The composition of amino acids and the physicochemical properties, such as charge and amphiphilicity. Generally, the positive charge of AMPs^1^ enables selective interaction with anionic bacterial membranes, while their hydrophobic regions facilitate engagement with the hydrophobic core of these membranes [105].
• **Sequence-level**. Sequence length, distribution (position), and motifs. For instance, the length of the AMPs is usually small (6–100 amino acids) [111].
• **Structure-level**. Specific structural characteristics are more likely to be associated with AMPs, such as α-helical, β-sheet, lasso, and cyclic [107], [112], [113], [114], [115].

Over the past period of time, researchers have identified and developed novel AMPs based on these enlightenments. However, the resultant methodologies have notably exhibited limitations in throughput and efficiency. AI-based methods, characterized by their data-driven trait, facilitate the capture of a broader range of data features, significantly accelerating the design of AMPs. Fig. 1 provides an overview of AI-driven AMP design. AI encompasses three fundamental components: input data, models or algorithms, and output results, denoted as X, f(.), and Y, respectively. At its core, it operates on the principle of transforming input data through algorithmic processing to generate output results, succinctly represented by the formula Y=f(X). For instance, the antimicrobial activity (Y) of an input sequence (X) can be determined by passing it through an AI-guided model (f(.)). Generally, AI consists of two parts, training and inference. The training phase entails the systematic optimization of a model’s parameters on a training dataset by utilizing algorithms such as stochastic gradient descent to minimize a predefined loss function, which quantifies the discrepancy between the model’s predictions and the ground truth. The inference phase capitalizes on the model’s previously learned parameters to infer outputs from new inputs, achieving the desired learning purpose, such as identification and generation. To enhance generalization and address overfitting and bias–variance tradeoffs, AI models incorporate techniques such as minimal empirical risk optimization, regularization, data augmentation using domain-invariant transformations, cross-validation with stratified sampling, and ensemble methods. A well-designed AI model requires exceptional performance, robust generalization, interpretable reasoning, ethical compliance, and scalable adaptability across diverse contexts.

**Fig. 2:**
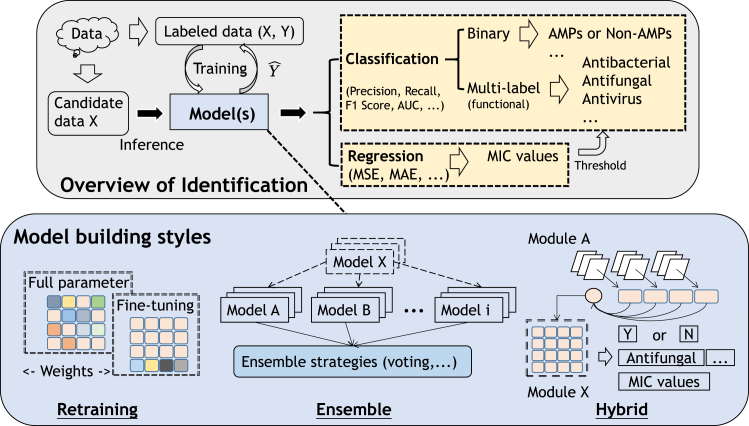
Overview of identification.

AI’s efficient framework makes it highly applicable across various fields, including AMP exploration. As illustrated in Fig. 1, constructing an AI model for AMP design begins with collecting relevant biological data and extracting representations to serve as inputs. Here, we summarize the main public AMPs’ databases [109], [116] in Table C.1 Appendix C. The five representational types are summarized by Wan et al. [117], including global descriptors (include sequence composition, structural features, and physicochemical properties) [118], [119], [120], [121], [122], [123], [124], sequence-based representation [125], [126], graph-based representation [127], [128], [129], 3D representation [130], [131], [132], and data-driven representation (AI-based automatically learning features) [133], [134], [135], [136]. After training on these represented data, the model can generate core predictions to expedite novel AMP discovery. Table C.2 Appendix C presents major AI-based methods dedicated to advancing AMP development.

In recent years, AI-based models have been applied to AMP design, which can be summarized into two levels: identification and generation. Identification emphasizes determining whether a given peptide sequence exhibits antimicrobial activity, encompassing tasks such as classification and regression. This foundational step provides critical insights into sequence-function relationships and serves as a prerequisite for the subsequent generative level. At the generative level, AI-guided models are employed to design novel AMP candidates de novo, creating sequences with potential therapeutic properties from scratch. This is a process where sequences go from nothing at all, and hence often requires iterative validation with multiple identification models to ensure the generated candidates meet desired activity, specificity, and safety criteria. In the following sections, we will delve into each of these levels, exploring their methodologies, advancements, and limitations.

## 3 Identification-oriented

Identification refers to distinguishing whether a given sequence possesses antimicrobial activity. Over the past few decades, AI-based identification methods for AMP design have undergone significant development. The basic tasks of identification are classification and regression, as shown in Fig. 2. The former directly maps the inputs into distinct categories, e.g., non-AMPs and AMPs, while the latter calculates the regression values of the inputs, e.g., the prediction of minimum inhibitory concentration (MIC) values, which not only reflect whether it is an AMP by setting a threshold but also indicate its antimicrobial effectiveness. Based on our investigation of AI-based methods for AMP identification, we summarize three model-building and design styles: retraining, ensemble, and hybrid.

### 3.1 Retraining models

Retraining existing models on new datasets is essential for maintaining model accuracy in adapting to new tasks with specified conditions or scientific insights. First, retraining ensures models remain accurate and effective over time, particularly in dynamic environments where data evolves and new discoveries emerge. For instance, as peptide sequences continue to be identified as AMPs, enriching the database, retraining allows models to incorporate the latest findings, thereby enhancing predictive capabilities and ensuring alignment with current scientific knowledge. Second, retraining serves as one of the foundations of *transfer learning*, often excelling in new tasks, such as identifying AMPs. To be well-compatible with new tasks, designers typically need to select appropriate representations according to task characteristics and modify the model to fit these requirements precisely, like a glove. There are two specific modes of this model modification: full parameter training and fine-tuning.

Full parameter training refers to the process of training a model by updating all of its parameters based on the training data. It allows models to be thoroughly optimized for specific scientific tasks and datasets. It is particularly necessary when the target task significantly differs from the original pre-training task or when the available data provides novel insights that the model needs to capture comprehensively. For instance, Sun et al. [137] transfer the sequence-based data into the graph-based data to retrain a two-layer GCN model to identify AMPs. They tested that a two-layer GCN can achieve the optimal performance, while too many GCN layers caused the model to be over-smoothing. During training, all model weights were updated, and the activation function, window size, convolution size, learning rate, loss rate, and training epoch were set to ReLU, 15, 200, 0.01, 0.5, and 150, respectively. Lee et al. [138] retrain SVM-based classifiers to investigate α-helical AMPs and the interrelated nature of their functional commonality and sequence homology. Specifically, they employed the L2-norm and trained three SVM classifiers with three distinct kernels, linear, polynomial, and radial basis function (RBF). Similarly, all weights were updated during training, and the hyperparameters were optimized via a grid search strategy. Through testing and evaluation, they recommended adopting the linear kernel. This recommendation is attributed to its superior performance and interpretability, coupled with the fact that the performance improvement offered by non-linear kernels proved insufficient. Fine-tuning refers to the process of adapting a pre-trained model to a specific task or domain by making minor adjustments to its parameters. This process enables the model to learn specific patterns and features relevant to the target task, thereby improving its accuracy and effectiveness. It allows models to leverage knowledge from pre-training while being customized for specific applications. Fine-tuning bridges the gap between general-purpose pre-trained models and the complex requirements of scientific tasks without requiring extensive computational resources or large datasets. For instance, large language models (LLMs) like BERT, with numerous parameters, are well-suited for fine-tuning in downstream tasks such as AMP identification [139]. Wang et al. [140] first trained an AMP-centric language model as the foundation model, called AMP-GPT, on a dataset of peptides extracted from UniProt [141]. Subsequently, they fine-tuned AMP-GPT to develop two submodels, AMP-Prompt and AMP-MIC. AMP-Prompt adopted a contrastive prompt tuning strategy by initializing the prompt embedding layer based on the word embedding of AMP-GPT. Only the parameters of this layer were updated, while the parameters of AMP-GPT were held fixed.AMP-MIC fine-tuned the AMP-GPT on the MIC datasets of three bacterial species, namely *S. aureus*, *E. coli*, and *P. aeruginosa*.

Although retraining offers simplicity and efficiency in operational implementation, it still has limitations. A single model that performs exceptionally well in only a few fields makes it difficult to fit perfectly into newly migrated problems. Ignoring the particular features of the new situation will often lead to overfitting and poor generalization ability.

### 3.2 Ensemble frameworks

An ensemble framework integrates multiple distinct models with an ensemble strategy, such as boosting and voting, to enhance prediction accuracy by coordinating multiple inferred results. These models can be of the same type (homogeneous) or different types (heterogeneous). The benefit of ensemble frameworks lies in harnessing the collective knowledge of multiple models to achieve superior performance compared to individual models, which are often constrained by their inherent biases and variances. In particular, ensemble models exhibit greater stability than individual models, as they are less susceptible to minor fluctuations in training data, which is particularly crucial for advancing scientific discovery, including AMP discovery. For instance, Ma et al. [111] implemented three models, ATT, LSTM, and BERT, for mining human gut microbiomes. By setting the unanimous voting strategy, they recognized 2349 sequences as candidate AMPs. Among these candidates, 216 were chemically synthesized, and 181 showed antimicrobial activity. Chen et al. [142] followed the same pipeline to mine the global marine microbial database and identified 121 unique candidate AMPs, of which 10 novel AMPs are successfully validated. Similarly, based on the same ensemble method, Xu et al. [143] mined 27 candidate AMPs from sludge and successfully synthesized 25 peptides among them. As a result, 21 peptides with antibacterial activity are experimentally verified against 4 strains.

The ensemble paradigm demonstrates notable efficacy in augmenting model generalization capability and operational robustness through the systematic integration of diverse constituent learners. This methodological framework capitalizes on the complementary strengths of heterogeneous base models, effectively mitigating individual model biases while enhancing collective decision-making through sophisticated aggregation mechanisms. By strategically amalgamating predictions from multiple learners, the ensemble architecture substantially reduces variance-related errors and improves resistance to data distribution shifts, thereby achieving statistically significant performance elevation compared with singular modeling approaches. Although this approach collects opinions is of wide benefit, it still has limitations. First, multiple models working together means more computational overhead. Second, the ensemble methods are very dependent on the strategy, which has limited effectiveness. For instance, the unanimous voting strategy greatly reduces the output space, thus missing some potential AMPs. In summary, the choice of ensemble technique should align with the specific task, data representations, and available computational resources to maximize benefits.

### 3.3 Hybrid building

Beyond directly retraining existing classical models or constructing ensemble frameworks, some designers propose novel architectures tailored to the unique challenges and data characteristics of AMP identification. A common approach is to combine modules^2^ from different existing models to rebuild a new architecture. The ensemble framework focuses on how to select or combine outputs from different models, whereas hybrid building focuses on integrating modules with diverse functionalities into a novel architecture. Each module fulfills its specific objective and functionality. Through mutual splicing and sequential cooperation, these modules collectively constitute a robust model. Not as compartmentalized and independent as the ensemble methods, the hybrid approach aims to embody all the strengths in one entity. For instance, iAMP-CA2L [144] is a deep neural network architecture combining CNN, BiLSTM, and SVM. The CNN part extracts amino acid sequence features effectively via feature mapping, the BiLSTM part captures contextual sequence features, and the SVM part is responsible for classification. Different modules assume distinct responsibilities. Li et al. proposed AMPlify [145], an attentive deep learning model for AMP identification, which applies two types of attention mechanisms layered on a BiLSTM layer. The Bi-LSTM layer encodes positional information from the input sequence, while one attention layer refines sequence representation using multiple weight vectors, and the other generates a summary vector by learning contextual information from the previous layer.

Hybrid approaches allow for the integration of diverse module capabilities, thereby creating a more versatile and comprehensive model that leverages the advantages of each constituent component. This amalgamation offers the potential for performance improvements and innovative solutions tailored to specific tasks. However, due to the necessity of integrating the modules as a cohesive whole, it is typically feasible to integrate only a limited number of individual elements rather than combining an extensive array of models, such as the dozen or more utilized in ensemble methods. Furthermore, the design of novel architectures or hybrid models demands substantial expertise and involves intricate design decisions.

To summarize, AI-based approaches enable the systematic exploration of a more profound feature space, thus capturing intricate relationships between sequences, distant source correlations, and salient features pertinent to identification. Notwithstanding the aforementioned distinctions among the three model types, several common limitations persist.

### 3.4 Challenges and limitations

Although there has been a proliferation of AI models designed for identifying AMPs, numerous challenges and dilemmas remain. We present a systematic summary of these issues across three dimensions, including task, data, and model.

**Task**. In general, data-driven and end-to-end models tend to understate the problem’s complexity, thereby neglecting the exploration of the intrinsic mechanistic nature of the task. Here, we summarize two points: the similarity trap and the absence of granular focalization. The similarity trap refers to the barriers to solution transferability, manifested through over-reliance on prior experiences and established frameworks while neglecting the embeddedness of problems within unique contextual ecosystems. Superficially analogous tasks often conceal divergent core mechanisms, necessitating context-driven solution reformulation, even in small aspects. For instance, although highly accurate structural prediction models emerge one after another, unfortunately, a single predicted structure (or conformation) may not sufficiently represent a given peptide because the short peptide’s conformational flexibility [117], [146], [147], [148], [149]. Therefore, the methods that rely on predicting structures to discover macromolecular proteins, such as enzymes, might not be quite suitable for identifying AMPs. The absence of granular focalization refers to the tasks constrained by broad generalizations and superficial assessments, while lacking systematic exploration into the essential and underlying mechanisms. As a result, it remains a niche area, with significant challenges to be overcome for broader applications. Synergistic mechanisms and chemical modifications are two classical archetypes. For example, many works have revealed that naturally co-occurring AMPs with distinct functions can synergize together [150], [151], [152], [153], but few AI-guided models directly explore or predict synergistic mechanisms among AMPs. The complexity of this problem and the difficulty of modeling constitute one aspect, while the other is the lack of necessary synergistic data. It is beneficial to enrich relevant data by collecting more measurement feedback about the synergisms among AMPs in vivo, rather than merely testing MIC values of individual components in vitro [154]. Moreover, although chemical modification is one of the important strategies to enhance antibacterial activity or improve stability, such as lipidation and N-terminal acetylation, current AI-guided approaches are generally confined to the 20 basic amino acids and rarely directly contribute to chemical modification.^3^ For instance, in the context of natural language models (NLMs) applied to AMP design, the vocabulary typically only consists of the 20 basic natural amino acids [111].

**Data**. The quality and quantity of training data are paramount to the model’s ability to learn effectively and generalize well, directly impacting its predictive accuracy and robustness. While publicly available datasets (Table C.1) exist for designing AMPs, their size is limited compared to extensive datasets available in computer science, due to the cost associated with wet labs. For instance, the classical computer vision dataset ImageNet contains over 14 million labeled samples [156], whereas APD3 [157] has only 5099 peptides for AMP designing. Furthermore, three limitations we have concluded in terms of AMPs’ data. First, specific data is scarce and noisy. While wet experimental validation is the sole criterion for discriminating whether a peptide is an AMP or not, not all AMPs have MIC data. Meanwhile, due to the varying environmental standards of wet labs, MIC data has noise, posing a challenge in the regression tasks. Second, coarse-grained. Antimicrobial activities, such as antibacterial, antifungal, and antivirus, are highly prevalent, yet there is a dearth of individual-level anti-labels, such as anti-*E. coli* or anti-*S. aureus*. Although datasets specifically designed to provide MIC values, such as GRAMPA [158], record more targeted information, they are outdated due to stagnant updates. Unfortunately, even though AMPs are broad-spectrum, they are not effective against all bacteria and still exhibit specificity. For instance, Ma et al. demonstrated their predicted AMP $c_{\text{\_}}AMP67$ could effectively destroy three strains of *K. pneumoniae* (NK04047, NK06129, and NK08334) but had little effect on NK01067 [111]. Therefore, coarse-grained categorization prevents researchers from knowing exactly which species a known AMP is effective against, which is not conducive to experimental replication and validation. There remains a need to develop more fine-grained works, such as the microbial strain-specific AMP Identification [159]. Third, negative data. While it is relatively easy to access several AMPs’ databases as summarized in Table C.1, there is a notable absence of specialized databases for non-AMPs, which is one of the major dilemmas in this field. Researchers often collected unlabeled samples as negative data, for example, a peptide without certain keywords related to ‘antimicrobial’ from UniProt as a negative one. However, this approach potentially leads to false-negative data that interfere with, or even mislead, AI models trained on them. Several strategies have been employed to enhance the selection of negative data, such as filtering similarity, label smoothing, and positive-unlabeled learning. These strategies are only temporary fixes rather than permanent solutions. All these methods are based on the common assumption that negative samples exist and are detectable. Unfortunately, it is not feasible to determine a negative sample since only wet-lab experimental validation can definitively determine whether a peptide is active or not, and it is impossible to test against all bacteria. Proving the positive is straightforward, but proving the negative is extremely challenging, which is referred to as unsurveyability [160]. Therefore, we cannot expect precise results from a vague categorization task. To this end, a change of perspective is needed to make the negative provable. Fine-grained classification could be a solution, focusing only on individual-level anti-labels as previously mentioned. For instance, experimental verifications can ascertain whether a peptide has anti-*E. coli* activity, simultaneously identifying positive and negative samples.

**Model**. High-quality data needs to be supported by a suitable model to enhance the effectiveness of AMP identification. However, AI models are not a panacea and still have limitations. First, they lack interpretability, especially for models within the deep neural network (DNN) framework. Their black-box attribute obscures the relationship between data and predictions, hindering the elucidation of intrinsic mechanisms. While various efforts have been made to enhance their explainability [161], [162], [163], [164], [165], [166], such as the attributional paradigm *Integrated Gradients* (IG) [161], these methods are post-hoc and primarily input-feature oriented. For instance, using physicochemical features inferred by explainable approaches and validated through wet experiments is a conventional strategy. However, providing specific attributional interpretations of the hidden space remains challenging, especially for deep learning models. Second, the absence of uniform criteria among models may result in inequitable comparisons. AI-based approaches exhibit significant variability in data quality, feature extraction, core algorithms, evaluation strategies, and metrics, with each study following its own protocols. Although several works [29], [167] have endeavored to conduct fair comparisons of various models for identifying AMPs, their comparative outcomes are inherently time-sensitive and diminish in relevance with the continuous emergence of novel methodologies. Additionally, the dynamic nature of training datasets presents another layer of complexity. Unlike static datasets in fields such as computer vision that are fixed and molded, antimicrobial peptide databases [157] are constantly evolving and expanding. Consequently, whenever a new model is designed, it is invariably trained on the latest version of the dataset. Simultaneously, authors often neglect to retrain baseline models on the newly collected datasets when benchmarking their new models. As a result, it is increasingly difficult to ascertain whether superior predictive performance is primarily due to the innovative design of the new method or the enhanced quality of the training data. Third, generalization. Generalization refers to a model’s ability to perform effectively on independent, unseen data rather than merely on the training data to which it has been fitted. This capability is crucial because the ultimate objective of AI-based approaches is to extrapolate from specific training instances to broader, general scenarios. Such models mitigate overfitting or underfitting to the peculiarities of the training set, thereby ensuring robust performance across a diverse range of inputs. Although several techniques, such as regularization and cross-validation, can enhance generalization, some critical aspects are often overlooked. For instance, the similarity between the training and the test dataset is frequently neglected in discussions. High similarity between these datasets may lead to seemingly high generalization performance. However, this setting is ultimately meaningless since it is vulnerable to subsequent unseen data that may exhibit significant variability. Fourth, reproducibility. Heil et al. [168] proposed three standards for computational reproducibility in 2021, including bronze, silver, and gold. Recently, Sidorczuk et al. [167] investigated the reproducibility of 26 models for AMP prediction and found that approximately 70% of the models represented non-reproducible work. Only 8 models met the minimal bronze standard. This study advocates for authors to provide exhaustive documentation regarding their models, with particular emphasis on training details. This comprehensive transparency would substantially enhance the reproducibility, thereby precluding futile endeavors, conserving valuable resources, and maintaining the momentum of scientific advancement in this domain.

### 3.5 Discussion

We advocate that rigorous architectural comparison demands identical training and test dataset deployments across all competitors, since only under such controlled conditions can genuine algorithmic superiority be isolated from data variance. Expanding the comparisons to fine-grained metrics is equally desirable, yet this inevitably obliges a complete retraining cycle for every model. However, it is an undertaking that is both time-intensive and computationally expensive. For this reason, few reviews satisfy the standard in its strictest form, and it is not the primary objective of our work. Therefore, no quantitative inter-model comparison is undertaken in this paper. Fortunately, some benchmark-oriented studies [139], [167], [169] prioritize uniform evaluation conditions and deliver precisely this level-playing-field benchmark. For instance, Gao et al. retrained the models on a unified AMP dataset and reported comprehensive metrics [139]. Rather than duplicating their effort, we therefore recommend directly referencing this type of benchmark-oriented literature.

## 4 Generation-oriented

**Fig. 3:**
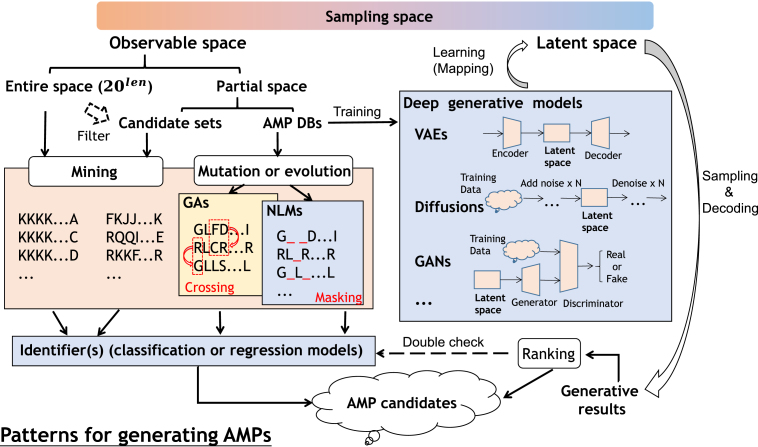
Overview of generation.

Building upon the capability to discern antimicrobial sequences, the subsequent challenge lies in the development of methodologies capable of generating a de novo repertoire of sequences endowed with antimicrobial potency. Beyond the determinations that are predicted from the identifying models, generative models facilitate the creation of novel data points that are consistent with the training data, such as a set of candidates with antimicrobial activity, reflecting a deeper understanding and insight into the probabilistic nature and structure of the data. This advancement would critically enhance the antimicrobial arsenal, offering novel therapeutic options and bolstering the ability to combat resistant pathogens. The generation process, involving the creation of new candidate sequences from scratch, is fundamentally centered on sampling. Specifically, it can be categorized into two aspects, sampling from the observable space and sampling from the latent space, as illustrated in Fig. 3.

### 4.1 Generation from observable space

Equipped with pre-trained identification models, exploring the observable space to check if a target sequence has antimicrobial activity is straightforward. Within this paradigm, peptide sequences in the observable space authenticated by the identifier(s) can be regarded as newly generated AMPs. For instance, the simplest approach is to screen the entire peptide sequence space. Huang et al. [170] mined the entire virtual library of peptides composed of 6–9 amino acids to identify potent antimicrobial peptides with an identifier. They synthesized the top-10 predicted peptides via solid-phase synthesis and tested their MIC values against *S. aureus*. All 10 peptides exhibited antimicrobial activities. This special antimicrobial peptide generation pattern is attributed to the limited discrete entire space, which benefits from the relatively short sequence lengths. In contrast, for continuous domains, employing exhaustive exploration through traversal is impractical. However, this approach, although effective, is not optimistic in terms of resource consumption for exploring the entire space, especially as the sequence length increases. Therefore, mining from the partial space is more desirable. Some candidate datasets with a greater likelihood of the presence of potential AMPs are favored [111], [142], [143], [171]. For instance, Ma et al. [111] mined AMPs from the human gut microbiome, while Chen et al. [142] mined the global marine microbial database. Both of them discovered novel AMPs successfully. The quality of this mining-driven mode hinges jointly on the candidate datasets and the identifiers employed. Selecting an inappropriate candidate dataset may prove futile. Furthermore, given that different identification models can produce varying results and no single model is universally superior, it is crucial to devise a suitable identification strategy prior to large-scale mining. Additionally, with the continuous emergence of new models, the results of mining-driven generation remain time-sensitive.

Another prevalent pattern draws inspiration from biological mutations and evolutionary processes. These approaches first sample sequences from AMP databases and then simulate the process of evolution or mutation by systematically altering the target sequences to generate novel candidates. Among these methods, genetic algorithms (GAs) [172], [173], [174] and neural language models (NLMs) [175], [176], [177], [178], [179] are particularly favored. GAs maintain a population of candidate solutions and iteratively evolve them through operations such as selection, crossover, and mutation, thereby efficiently exploring the solution space. For example, Porto et al. leveraged a genetic algorithm to explore the potential space of the guava peptide, *Pg-AMP1*, and generated the guavanin peptides, one of which displayed potent activity against Gram-negative bacteria [173]. NLMs employ a sophisticated mechanism involving masking and generation to comprehend and reconstruct sequences. They predict concealed tokens based on contextual information derived from unmasked tokens, thereby learning the nuanced relationships between amino acids and their surrounding environment. However, despite exhibiting clear goal-oriented behavior and facilitating efficient exploration of local spaces, these methods are inefficient in exploring the global space. Consequently, the diversity of sequences generated by such approaches is limited.

### 4.2 Generation from latent space

Different from the generative modes that rely on sampling from the observable space, some DNN-based models [180], such as variational autoencoders (VAEs) [181], [182], [183], [184], generative adversarial networks (GANs) [185], [186], [187], [188], and diffusion models [189], [190], [191], [192], [193], [194], generate novel candidates by sampling from the latent space. The latent space serves as a compact, continuous representation that encapsulates the intrinsic structure and variability of the training data, enabling the model to learn a more efficient and meaningful encoding of the data’s underlying features. Take VAEs as an example, by first mapping the input data to a lower-dimensional latent space and then decoding it back to the original data space, these models produce a wide array of outputs while maintaining coherence and relevance to the learned data distribution. Sampling from the pre-learned latent space, VAE-based models, such as PepVAE [181], LSSAMP [184], and PepCVAE [183], facilitate the generation of new samples analogous to the training data but with novel characteristics. In the case of GAN-based models, the generator learns to produce high-quality candidate sequences from the latent space through continuous correction and guided feedback from the discriminator. Lin et al. [185] synthesized 8 AMPs generated from their proposed GAN-based models, two of which exhibited broad-spectrum antibacterial effects and were effective against antibiotic-resistant bacterial strains, such as *S. aureus*. Diffusion-based models rely on adding noise to build the latent space, such as Gaussian noise, and then refining random noise into data samples by reversing a diffusion process. Finally, by sampling the latent space, the model can generate new elements that are similar to the training data. Based on this principle, Chen et al. proposed AMP-Diffusion [195], Qi et al. proposed CDiffusion-AMP [196], and Wang et al. proposed Diff-AMP [197] for AMPs generation.

### 4.3 Complete generative pipeline

We here summarize the complete generative pipeline, spanning from in silico generation to wet-lab validation, including six core steps, as follows.

• **Task anchoring**. In light of objective conditions, such as available computational resources and the accessibility of in-house datasets, researchers need to crystallize the task objective and to chart the entire implementation pathway. For example, generation from the latent space or generation from the observable space.
• **Data collection and representation**. AMP datasets are customarily assembled through the systematic integration of multiple repositories, thereby augmenting sample size. Concomitantly, a diverse repertoire of feature-extraction protocols has been elaborated to capture the signatures of these sequences. The corresponding databases and strategic feature-encoding schemes are catalogued in Appendix B for reference.
• **Model designing, training, and inference**. The model architecture should be tailored to the precise objectives of the task and the characteristics of the curated data. Some hyperparameters might inevitably rely on empirically informed choices to secure peak performance. Computational resources must be meticulously scheduled in advance to obviate superfluous expenditure.
• **Computational evaluation and filtering**. Generated candidates undergo multi-layered filtering: predictions for antimicrobial activity, novelty and diversity, and biological criteria. We summarize these evaluation and filtration strategies in Appendix A.
• **Wet-lab validation**. The ultimately shortlisted candidate sequences will undergo wet-lab validation via a curated suite of assays, including organisms and antimicrobial assay [111], [140], [173], [182], [185], [198], [199], hemolysis assay [111], [140], [170], [173], [198], plasma stability test [140], time-kill assay [140], [170], [173], membrane permeabilization [170], [173], [182], [200], cytotoxicity assay [111], [170], [182], [200], NMR experiment [173], [174], [201], CD spectroscopy [173], resistance assay [140], [170], [182], and in vivo activity test [140], [173], [174], [182], [199].
• **Feedback**. Two feedback types need to be accommodated. At the knowledge-base aspect, wet-lab outcomes are scrutinized to determine whether they corroborate established tenets, overturn prevailing dogma, or unveil previously unrecognized biological principles. At the modeling aspect, the wet-lab outcomes are channeled back into the pipeline, enabling reinforcement-driven retraining (reinforcement learning) that iteratively refines and evolutionarily advances the model.

**Fig. 4:**
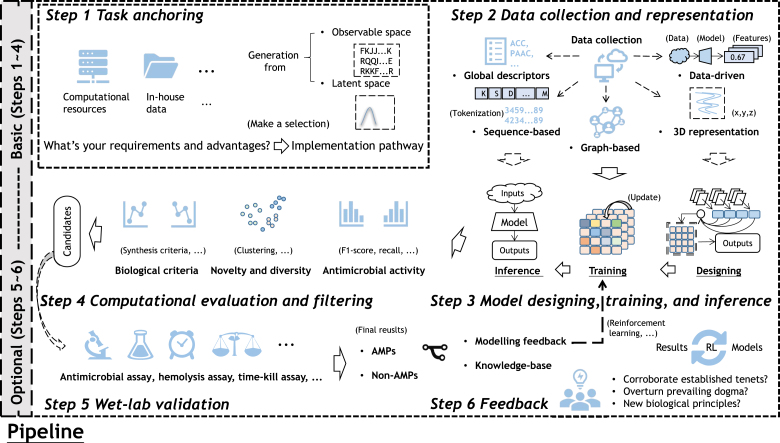
Overview of pipeline.

Fig. 4 shows an overview of the pipeline. The initial four steps constitute the foundational and uniform core of the pipeline; nevertheless, wet-lab validation is not universally furnished across the studies, especially in computation-oriented literature. Analogously, the reinforcement-cycle feedback invoked in Step 6 is also far from being a consistently implemented component of every modeling framework. A fully elaborated exemplar is therefore indispensable, as it affords a step-wise point from which to apprehend the complete pipeline in operation. To break the “bacteria-only” bottleneck of current AMP generators, Wang et al. [200] collected four datasets and extracted one-hot features to train their designed modeling framework PepDiffusion, which can generate antibacterial and antifungal peptides from the latent space. Then, they used three filtrations (classification, clustering, and coarse-grained MD simulations)^4^ to sift through the 600,000 generated peptide sequences, and thus selected 40 for wet-lab validations, including MIC determination assay, membrane permeabilization assay, drug resistance assay, hemolysis assay, cytotoxicity assay, and in vivo studies (Murine). The result shows that 25 filtered peptides exhibited antibacterial or antifungal activities. Among them, 5 had selective activity against specific fungal species, suggesting that these AMPs have limited universal activity.

### 4.4 Challenges and limitations

The generative models not only facilitate the generation of a substantial number of candidate sequences but also endeavor to minimize the inclusion of negative samples during the training phase, thereby effectively circumventing the influence of the ineradicable noise inherent in negative samples, as elaborated in Section 3. Nevertheless, in addition to sharing some common drawbacks with identification models, such as non-interpretability, they are also subject to several additional limitations.

**Duplicate candidates**. In contrast to the mining-driven generation mode, which does not produce duplicate candidates, generative models often yield multiple duplicate sequences. For example, Lin et al. [185] had to remove 1225 duplicate GAN-designed peptides before proceeding to experimental validation. Unfortunately, such repeated sequences are difficult to avoid, as generative models essentially compress the entire sequence space into the functional sequence space. Duplicates arise both from sampling the same elements in the latent space and from mapping different sample points from the latent space to the same sequence.

**Delayed and indirect feedback**. Wet experimental validation stands as the sole definitive method for identifying antimicrobial activity. However, this procedure is time-consuming and costly. Consequently, an intermediate step is frequently indispensable to refine the selection of candidates prior to experimental validation. This step typically involves supplementary identification models, such as those predicting the MIC values, to rank and filter the generated candidates. As a consequence, the selection is narrowed down to those sequences with the highest likelihood of antimicrobial activity, thereby enhancing the efficiency and accuracy of subsequent experimental validation. Nevertheless, this approach introduces an additional layer of influence from the identification models employed in the double-check, which interferes with the direct reflection of the intrinsic generative quality of the original generative models.

**Ambiguous metrics**. The pre-segmentation of the test dataset prior to the training phase enables the quantitative assessment of identification models through metrics such as precision, recall, and F1-score. In contrast, evaluating generative models is more intricate, as it aims to generate both high-quality and diverse samples. This complexity necessitates a combination of quantitative metrics to ensure the models’ effectiveness and utility. Meanwhile, due to the absence of a unified standard, the subjective nature of quality assessment in generative models introduces variability, as different evaluators have varying perceptions of the generated samples’ quality. Take diversity as an example, Wang et al. utilized *Levenshtein distance* [202] to evaluate the diversity of candidates generated by their VAE-based model LSSAMP [184], while Van Oort et al. employed the *Gotoh global alignment algorithm* [203] for diversity assessment.

### 4.5 Discussion

We here turn to a discussion of feature extraction, a common procedural component that is indispensable to both discriminative and generative paradigms. In Section 2, five feature extraction schemes are briefly introduced, following a comprehensive review [204]. We survey the specific implementation details of these five categories in Appendix B. Then, we investigate whether there exist some features that are unique to AMPs. To date, no feature has yet been devised specifically for this purpose. Researchers prioritize generalized protein representations, refraining from subjective weighting of individual features and instead adopting rigorous statistical strategies (e.g., covariance analysis [205]) for subsequent feature selection (dimensionality reduction). Conversely, several feature strategies that are commonplace in other protein prediction domains remain largely absent from AMP tasks. For instance, three-dimensional features. Although some studies extract three-dimensional features as AMP representations [206], such approaches are adopted far less frequently than in PDB (protein data bank)-centric prediction tasks such as protein–protein interaction site (PPIS) identification [131], [207]. Moreover, even when three-dimensional features are extracted, this representation does not serve as the sole input to models like sequence representations, but rather combines with numerous other distinct representations as inputs [206]. It is attributable to the fact that AMPs are short sequences, rendering their three-dimensional conformations inherently unstable and thus introducing substantial uncertainty. In summary, researchers have exercised considerable caution in incorporating three-dimensional features into AMP design tasks.

## 5 Flexible design

Enhanced by artificial intelligence, researchers can adaptively design AMPs from diverse perspectives, offering a rich tapestry of solutions and opportunities for innovation.

**Diverse perspectives**. Multifaceted analysis, which examines issues from diverse perspectives, is instrumental in enhancing our understanding and resolution of complex problems, thereby providing a significant impetus for the advancement of the AMP design. For instance, existing works have taken the perspective on the interaction with membranes [208], [209], molecular dynamics (MD) simulations [182], [210], [211], site prediction [212], and functional domains [174], [213]. Each view contributes uniquely to the advancement of AMP-based characteristics, highlighting the necessity of embracing diverse perspectives in the pursuit of novel and effective antimicrobial solutions. For example, MD simulation provides atomic-level details of AMPs interacting with lipid bilayers and other biological membranes. This elucidates the intricate mechanisms of how AMPs disrupt bacterial membranes while sparing cells, aiding in the design of more stable and effective peptides, and thus serving as a bridge between computational design and virtual experimental validation [182]. Various perspectives have jointly contributed to the flourishing of the process of AMP design, holding the promise of exploring the design-function space of AMPs at an increased rate and depth.

**Multipath**. Three crucial principal paths govern AMP development, mining, redesign, and de novo design. Each of them demonstrates distinct operational paradigms within the AMP discovery pipeline. Mining exploits existing databases or potential in-house datasets, such as marine microbial databases [142] and human gut microbiomes [111], to rapidly discover novel AMP candidates, accelerating the identification of peptides with specific antimicrobial properties. This primary data-driven mode accelerates candidate identification while minimizing experimental validation requirements, though constrained by existing sequence bias, enabling rapid preliminary screening of established antimicrobial scaffolds. Rational redesign enhances existing AMP performance through strategic modifications. This iterative process improves pharmacological parameters (such as target affinity, proteolytic stability, and cytotoxicity) while maintaining core antimicrobial motifs. Compared with mining, redesign is optimization-focused, typically building on known peptides, which makes the motives and demands more specific. De novo design utilizes generative architectures to explore uncharted chemical space, thus creating novel sequences with emergent antimicrobial mechanisms. This paradigm-shifting mode explores a vast space without explicit templating on existing sequences, yet requires rigorous validation of functionality and biosafety profiles. Although the distinction in protein design between redesign and de novo design is more nuanced than the dichotomy, since both of them incorporate functional motifs from natural sequence and structural elements to varying degrees in the training phase [204], de novo design demonstrates superior sequence diversity through enhanced exploration of potential sequence space. Together, these three paths accelerate discovery, enhance performance, and expand the therapeutic potential of AMPs.

**Multimodality**. The performance of AI models is typically enhanced by the quantity and quality of training data, as well as by the integration of multiple data modalities to deepen the model’s comprehension and interaction. Notin et al. concluded three core modalities for functional protein design, including sequences, structures, and functional labels [204]. Wan et al. further summarized five representational modalities based on them, including global descriptors, sequence-based representation, graph-based representation, 3D representation, and data-driven representation [117]. Each modality has its own strengths and limitations, contingent upon the specific tasks and data availability. For instance, global descriptors succinctly summarize peptide properties, which is particularly advantageous as it captures specific information relevant to the modeled properties. Compared with it, graph-based representations are better inputs for geometry-related tasks because they capture connectivity information. By integrating multiple data modalities, AI models enable a more comprehensive understanding and generation of information, leading to improving data complementarity, enriching semantic representation, reducing data dependency, and enhancing contextual understanding [214], [215], [216].

**Multistage**. The advent of diverse technological tools [120] and computational platforms [217] for peptide engineering has catalyzed a paradigm shift from singular-method approaches to sophisticated multistage design pipelines that synergistically integrate heterogeneous methodologies, thereby enabling the implementation of diversified design strategies with distinct objectives at sequential phases. For instance, target-constrained tertiary architectures generated via RFdiffusion [193] undergo inverse folding through ProteinMPNN [218] to probabilistically decode sequence ensembles, which are subsequently double-checked via ESMFold [74] to ensure sequence-structure congruence. Despite the potential of protein structure-function-guided design methods [219], [220], the inherent instability of AMPs poses a significant challenge due to their small size and flexible conformation [211], [221]. This instability impedes their practical application and necessitates the development of strategies to enhance their robustness. Another paradigmatic illustration lies in the synergistic collaboration between identification models and generative models. When generative models produce candidate sequences, there exists a critical need for subsequent multi-criteria discriminative screening through identification models. This iterative multistage cross-validation mechanism establishes a self-reinforcing feedback loop that significantly enhances model robustness by mitigating confirmation bias inherent in single-model approaches. Moreover, cutting-edge implementations (such as AMP-designer [140]) are increasingly integrating wet-lab experimental data with *reinforcement learning* frameworks to create closed-loop optimization systems. This feedback enables continuous model retraining, thereby progressively enhancing the physicochemical validity and biological relevance of generated sequences. Such hybrid computational–experimental pipelines have demonstrated particular efficacy in de novo protein design challenges where traditional purely in silico methods struggle with the combinatorial complexity of sequence-structure-function relationships. In summary, this multistage closed-loop paradigm enables continuous model refinement via retraining and multi-objective optimization, systematically improving robustness in identifying sequences that satisfy specific functional constraints while mitigating confirmation biases inherent in single-stage approaches.

## 6 Discussions

Numerous strategies have been developed to investigate novel AMPs. Bioassay-guided approaches, such as chromatography and fluorescence screening [222], demonstrate high precision yet are constrained by their time-intensive and costly characteristic. Empirical design, for example, enriching cationic or hydrophobic residues that are strongly associated with antimicrobial activity, holds merit but heavily relies on established knowledge, which results in bottlenecks in the scope of exploration. Due to these limitations, large-scale implementation remains impractical and ineffective. Traditional bioinformatics methods, such as BLAST (Basic Local Alignment Search Tool) [223], offer significant advantages in sequence comparison, potentially identifying AMPs. Despite its effectiveness, the flat filter style of this method restricts the diversity of its conclusions. In contrast, AI-based methods, capable of analyzing vast datasets with high accuracy and identifying complex patterns and trends, have effectively accelerated AMP design. However, this field is still in its early stages, and numerous challenges remain to be addressed and resolved. We summarize the challenges and prospects throughout our survey as follows.

### 6.1 Challenges

Vast peptide space, undesirable physicochemical properties, and unspecific mechanisms of action stall the discovery of novel AMPs. Meanwhile, the high cost of peptide synthesis as well as the generation of more industrial wastes hinder the implementation of translating AMPs as therapeutic drugs to treat infectious diseases [224]. Furthermore, although AMPs exhibit more favorable pharmacodynamics than conventional antibiotics in terms of preventing resistance evolution, bacteria can still evolve resistance to AMPs. For instance, Sinorhizobium meliloti has been investigated to evolve a peptidase that protects it from the harmful effects of AMPs [225]. These challenges underscore the intricate nature of AMP development and emphasize the need for more in-depth and sustained exploration. AI-guided methods have emerged as a promising pipeline to accelerate this process by efficiently identifying potential AMP candidates from large libraries. Nevertheless, artificial intelligence is not a panacea. The limitations in terms of identified and generative models for designing AMPs have been discussed in Sections 3.4, 4.4, respectively.

In addition to the aforementioned limitations, AI-guided technologies are constrained by inherent restrictions, termed *impossibility theorems*. These theorems are classified into five mechanism-based categories: *deduction, indistinguishability, induction, tradeoffs*, and *intractability* [226]. For instance, the renowned *Gödel’s Incompleteness* theorems [227] fall under deduction, while the well-known *No Free Lunch* (NFL) theorems [228], [229] are categorized into induction. These impossibility theorems provide crucial guidance and caution on building AI systems for AMP design. Take the induction impossibility theorems for an example, many AI-guided approaches, such as statistical learning and deep learning, rely on inductive reasoning, which leads to Goodman’s problem [230], underscoring that predictions of AMPs are not logically guaranteed. Specifically, one of the prediction biases stems from the implicit assumptions embedded in AI algorithms, such as the assumption of independent and identically distributed (i.i.d.) data. This assumption posits that data samples are drawn independently from the same probability distribution. However, in the scenario of AMP design, candidates deviate from this ideal condition, thereby potentially reducing the accuracy of model predictions. Furthermore, AI-guided methodologies tend to converge to sequence clusters that resemble those in the observed datasets and generally lack the capacity to explore entirely novel sequence clusters. This phenomenon accentuates the inherent limitations of AI in terms of de novo exploration and making groundbreaking discoveries, as AI systems predominantly rely on pre-existing data and established patterns. Therefore, it is essential to integrate multi-scale approaches and specialized knowledge to collectively drive its development. Subsequently, as novel data continuously accumulates, the quality driven by AI can be continuously improved, thereby deducing more valuable AMP candidates.

### 6.2 Prospects

The various challenges inherent in antimicrobial peptide design impel persistent exploration and resolution. While presenting formidable hurdles, they simultaneously act as catalysts for innovation and discovery, continually giving rise to numerous opportunities for advancement. Concurrently, as datasets continue to be enriched and expanded, models are iteratively refined, and knowledge bases are augmented, the design of novel AMPs will be significantly promoted. We summarize three aspects for future research in AI-accelerated AMP design, including finer-grained exploration, model-driven data enrichment, and model enhancement.

**Finer-grained explorations**. AMPs exhibit broad-spectrum antimicrobial activity. Nevertheless, emerging evidence has revealed previously unrecognized target-specific activity patterns [231], [232], [233]. Studies on these specificity mechanisms constitute a critical strategy for enhancing drug targeting capabilities [234], [235]. Systematically delineating the mechanistic interplay between AMPs and diverse biological targets may guide the development of more potent systemic applications. Similarly, investigating synergistic interactions among AMP combinations represents a potential approach to augment antimicrobial efficacy and mitigate resistance risk. Additional promising research avenues include strategic chemical modification for improving stability, systematic elucidation of fundamental biophysical properties, and conditional generation with finer control for precision design. Without granular mechanistic studies, unresolved barriers will consistently impede clinical advancement. For instance, the current restriction of AMPs to topical formulations rather than systemic administration represents a substantial obstacle to their implementation as viable pharmaceutical agents.

**Model-driven data enrichment** The paucity of high-resolution training datasets constrains AI-assisted AMP design. For instance, the scarcity of data regarding AMP synergies precludes the development of models capable of discerning novel cooperative relationships among AMPs. This limitation underscores the imperative for specialized data acquisition aligned with model-driven requirements, a trajectory that reflects the escalating importance of interdisciplinary collaboration. Biologists, in partnership with modeling specialists, can prioritize the collection of targeted data to enhance model functionality and robustness. For example, rather than relying exclusively on in vitro MIC testing of individual components, they can measure AMP synergisms both in vivo and in vitro. Although this collection demands significant resources and temporal investment, it represents a crucial foundation for sustaining high-quality research initiatives over the long term. Another example concerns chemical modification. Enriching training datasets with non-standard peptides instead of only 20 standard amino acids will significantly expand the potential design space and enhance model generalizability. Incorporating these tailored libraries into training datasets represents a promising avenue for future progress. In parallel, the integration of multi-scale data into unified modeling architectures is gaining prominence, exemplified by multimodal models. As a result, dataset enrichment now extends beyond increasing data volume (depth) to encompass a broader range of data types (breadth).

**Model enhancement** AI models function as intermediaries between observational data and predictive insights alongside continuous refinement and advancement. Two trends in model enhancement may catalyze the accelerated design of AMPs: scalable generalization and refined specialization. The scalable generalization benefits from the scaling laws [236], which delineate the quantitative relationships between model performance and factors such as model size, dataset size, and computational resources, typically observed as power-law relationships. Additionally, during the accumulation of these resources, a phenomenon termed “emergence” [237], [238] occurs, wherein new capabilities or characteristics suddenly manifest in a system following gradual and seemingly minor alterations. For instance, the integration of multi-scale data exemplifies how multimodal models excel in capturing heterogeneous data across scales, enabling comprehensive representation learning and cross-modal synergy. Similarly, the *Mixtral of Experts* (MoE) models [239], [240], [241], [242] enhance model robustness through dynamic expert routing for adaptive computation. These large-scale models offer a macroscopic and comprehensive perspective to guide the design of AMPs. In parallel, functional AI agent system [243], [244] conveniently serve end-to-end applications [245]. This complete pipeline will predict the results of novel AMPs from de novo design through all pre-clinical validations for reference, for instance, toxicity concerns, delivery systems, and immune response. The refined specialization refers to optimizing the model for specific tasks to enhance its learning and inference capabilities. For instance, fine-tuning [246], [247], [248], [249] can adapt a pre-trained model to a specific task or dataset by further training, thereby enabling efficient transfer learning and improved performance. Furthermore, purely data-driven models exhibit suboptimal performance when dealing with scarce or noisy data. By incorporating physical constraints, the robustness and interpretability of the model are enhanced. A prime example is *IC-Light* [250], which incorporates the physical principle of consistent light transmission. With this consistency, it enables uniformly handling various data sources and facilitating a physically grounded model behavior, hence guaranteeing accurate light modification while preserving intrinsic properties. These physics-informed models [251], [252], [253] improve the validity and reliability of predictions and exhibit proficiency in conducting finer-grained data analysis and learning for specific tasks. Similarly, in the foreseeable future, a proliferation of models incorporating biological principles is anticipated to emerge, for example, equipped with biophysics principles to provide the necessary inductive knowledge to extrapolate beyond the observable samples.

Artificial intelligence catalyzes antimicrobial peptide design. By combining computational efficiency with the biological environment and bridging computational innovation with practical therapeutic solutions, this transformative paradigm not only accelerates the design cycle but also expands the chemical and functional diversity of AMPs, representing a cornerstone for next-generation antimicrobial agents tailored to combat evolving pathogens with escalating antimicrobial resistance. However, despite the immense development potential and promising applications of AMPs, it is imperative to proceed with caution and avoid the pitfalls of overeager advancement, as Lazzaro et al. suggested in 2020 [154], “If AMPs are to be used clinically, it is crucial to understand their natural biology in order to lessen the risk of collateral harm and avoid the crisis of resistance now facing conventional antibiotics”.

## CRediT authorship contribution statement

**Yongqiang Liu:** Writing – review & editing, Writing – original draft, Visualization, Validation, Supervision, Resources, Project administration, Methodology, Investigation, Formal analysis, Data curation, Conceptualization. **Jie Hu:** Validation, Investigation, Data curation. **Ning Zhang:** Validation, Investigation, Data curation. **Yinqi Bai:** Writing – original draft, Validation, Formal analysis, Data curation. **Yuan Yao:** Writing – original draft, Validation, Funding acquisition. **Gaoxiang Chen:** Writing – review & editing, Writing – original draft, Validation, Project administration, Funding acquisition, Conceptualization.

## Declaration of competing interest

The authors declare that they have no known competing financial interests or personal relationships that could have appeared to influence the work reported in this paper.

## Figures and Tables

**Table C.1:** AMPs’ Databases^a^.

Database	Description	Last updated^b^	URL	Reference(s)
CyBase	Cyclotides. Database of proteins that possess a cyclic backbone in which the N and C termini have been joined.	2025.04	https://www.cybase.org.au/	[114], [115]

APD3	Including natural AMPs from the six life kingdoms, synthetic, and predicted AMPs with 25 activities.	2025.01	https://aps.unmc.edu/	[157], [289], [290]

dbAMP	Including predicted AMPs and natural AMPs with 23 activities.	2024.11	https://awi.cuhk.edu.cn/dbAMP/index.php	[291], [292]

DRAMP	Including sequences, structures, activities, physicochemical, patent, clinical, and reference.	2024.11	http://dramp.cpu-bioinfor.org/	[293], [294], [295], [296]

AMPSphere	A collection of AMPs from the global microbiome, containing 863,498 distinct sequences, clustered into 10,715 AMP families with at least 7 sequences each.	N/A (2024.07)	https://ampsphere.big-data-biology.org/about	[171]

MBPDB	Includes all known bioactive peptides derived from milk proteins from any species.	2024.06	https://mbpdb.nws.oregonstate.edu/about_us/	[297], [298]

AMPDB	A feature-rich knowledge base on AMPs that assimilate information from 30 databases with 88 functional activities.	2023.04	https://bblserver.org.in/ampdb/	[299]

$CAMP_{R4}$	Including predicted AMPs and natural AMPs on sequence, structure, activity, source organism, target organisms, protein family descriptions, modifications, and links.	N/A (2023.01)	https://camp.bicnirrh.res.in/index.php	[300], [301], [302], [303], [304]

MEGARes	Hand-curated resistance genes for antimicrobial drugs, biocides and metals, incorporating 4 data sources.	2022.09	https://www.meglab.org/megares/	[305], [306], [307]

B-AMP	A structural and functional repository consisting of 5766 AMPs from natural and synthetic sources.	2022.08	https://b-amp.karishmakaushiklab.com/index.html	[308], [309]

DBAASP	Including ribosomal, non-ribosomal, and synthetic peptides that show antimicrobial activity as Monomers, Multimers, and Multi-Peptides.	N/A (2021.01)	https://dbaasp.org/home	[310], [311], [312]

AHTPDB	Database of experimentally validated Antihypertensive peptides, containing 5978 entries of about 1694 unique peptides.	N/A (2015.01)	https://webs.iiitd.edu.in/raghava/ahtpdb/	[313]

CancerPPD	Database of experimentally verified anticancer peptides and proteins, containing 3491 entries.	N/A (2015.01)	https://webs.iiitd.edu.in/raghava/cancerppd/	[314]

starPepDB	An integrated graph database holding bioactive peptides and metadata from a large variety of biological data sources.	2014.12	https://mobiosd-hub.com/starpep/	[315], [316]

ParaPep	Database of experimentally validated anti-parasitic peptides, containing 863 anti-parasite peptide entries, which have been tested against 12 different types of parasites.	N/A (2014.06)	https://webs.iiitd.edu.in/raghava/parapep/	[317]

YADAMP	Particular attention on short alpha helix peptides interacting with cell membranes.	2013.03	http://yadamp.unisa.it/	[318]

a The date of the latest update and verification is April 17, 2025.

b The content within the parentheses indicates the publication date of the latest literature.

**Table C.2:** AI-guided methods for designing AMPs^a^.

Reference	Year	AI-module(s)	Task	Wet-lab	Web Server	Open-source codes
EvoGradient [277]	2025	CNN, LSTM, ATT, and TF	Classification	Y	N	Y
Shen et al. [280]	2025	CNN, LSTM, and BERT	Classification	Y	N	N
TranSAFP [155]	2025	CNN, LSTM, ATT, and GRU	Classification	Y	N	N
AMP-Designer [140]	2025	TF, ATT, and RNN	Generation	Y	N	Y
Zhang et al. [174]	2025	GA and KNN	Generation	Y	N	N
Gao et al. [201]	2025	ESM and GA	Generation	Y	N	Y
PepDiffusion [200]	2025	TF, VAE, and Diffusion	Generation	Y	N	Y
Chung et al. [261]	2024	CNN, BiLSTM, RF, and TF	Classification	N	N	Y
dmSLAY [209]	2024	RF, SVM, and DT	Classification	Y	N	Y
iAMP-Attenpred [282]	2024	CNN, BERT, BiLSTM, and ATT	Classification	N	N	Y
AGRAMP [323]	2024	RF	Classification	Y	Y	N
Diff-AMP [197]	2024	Diffusion and CNN	Generation	N	Y	Y
AMP-Diffusion [195]	2024	Diffusion	Generation	N	N	N
HydrAMP [198]	2023	VAE	Generation	Y	Y	Y
Teimouri et al. [324]	2023	SVM and LR	Classification	N	N	Y
AMP-BERT [284]	2023	BERT	Classification	N	N	Y
Cao et al. [187]	2023	GAN and BERT	Generation	Y	N	Y
Huang et al. [170]	2023	XGBoost, CNN, RF, and LSTM	Classification	Y	N	Y
DeepAFP [278]	2023	BERT, CNN, and BiLSTM	Classification	N	N	Y
iAMPCN [279]	2023	CNN	Classification	N	N	Y
MBC-Attention [262]	2023	CNN and ATT	Classification	N	N	Y
Ma et al. [111]	2022	ATT, LSTM, and BERT	Classification	Y	N	Y
sAMP-PFPDeep [325]	2022	CNN	Classification	N	N	Y
LSSAMP [184]	2022	VAE	Generation	Y	N	Y
AMPpred-EL [268]	2022	LightGBM and LR	Classification	N	N	N
Sun et al. [137]	2022	GCN	Classification	N	Y (error: 404)	N
AMPlify [145]	2022	BiLSTM and ATT	Classification	Y	N	Y
MLBP [281]	2022	CNN and RNN	Classification	N	N	Y
Das et al. [182]	2021	LSTM, WAE, and CLaSS	Generation	Y	N	Y
Capecchi et al. [179]	2021	RNN	Generation	Y	N	Y
PepVAE [181]	2021	VAE	Generation	Y	N	N
Wang et al. [178]	2021	LSTM and BiLSTM	Generation	N	N	N
AMPGAN v2 [186]	2021	BiCGAN	Generation	N	N	Y
iAMP-CA2L [144]	2021	CNN, BiLSTM, and SVM	Classification	N	N	Y
Zhang et al. [283]	2021	BERT	Classification	N	N	Y

a Abbreviations: Convolutional Neural Network (CNN) [326], Bi-directional Long Short-Term Memory (BiLSTM) [327], Random Forest (RF) [328], Transformer (TF) [329], Attension (ATT) [330], Long Short-Term Memory (LSTM) [331], Gated Recurrent Unit (GRU) [332], ESMFold (ESM) [74], Bidirectional Encoder Representations from Transformers (BERT) [333], Support vector machine (SVM), Decision Tree (DT) [334], Genetic Algorithm (GA) [335], Fuzzy k-nearest Neighbor (fuzzy k-NN) [336], Variational Autoencoder (VAE) [337], Logistic Regression (LR) [338], Generative Adversarial Network (GAN) [339], [340], EXtreme Gradient Boosting (XGBoost) [341], Light Gradient-Boosting Machine (LightGBM) [342], Graph Convolutional Network (GCN) [343], Wasserstein Autoencoder (WAE) [344], Recurrent Neural Network (RNN) [345], Bidirectional Conditional Generative adversarial Network (BiCGAN) [346].

## Appendix A Computational evaluation and filtration types of AI-driven AMP design

Between the candidate sequences generated from generative models and the subsequent execution of wet-lab validation lies a critical intermediate phase, computational evaluations and filtrations. Based on a systematic survey of the literature, beyond basic operations like deduplication, we consolidate three evaluation and filtration types, predictions for antimicrobial activity, novelty and diversity, and biological criteria, as follows.

**Predictions for antimicrobial activity.** Antimicrobial activity is the most important property of AMPs. Therefore, it is essential to evaluate whether candidate sequences possess this property, as follows.

• Identification models (classifiers) with statistical metrics [140], [182], [183], [185], [186], [197], [198], [200]. As the most frequently employed computational validation strategy, an identification model reassesses each generated candidate peptide. Sequences must satisfy a comprehensive suite of statistical criteria derived from the four cardinal indices of the confusion matrix, true positives (TP), true negatives (TN), false positives (FP), and false negatives (FN), and their derivatives, such as F1 score, Recall value, MCC (Matthews Correlation Coefficient); only those classified as antimicrobially active are permitted to proceed the next filtration.
• Molecular dynamics (MD) simulations [182], [198], [199], [200]. MD simulations are implemented using the predicted structures of the peptides in the presence of lipid membranes to test their binding mechanisms. Candidates with antimicrobial properties can align parallel to and infiltrate deeply within the membrane [28], [254]. For instance, GROMACS [255] can be employed to execute MD simulations: negative distances denote that the peptide remains solvated and distant from the membrane surface, consistent with a non-antimicrobial phenotype, whereas positive distances signify membrane contact or insertion, indicative of antimicrobial activity.

**Novelty and diversity.** The principal merit of generative models lies in their capacity to yield candidate peptides that depart from the extant antimicrobial repertoire. Consequently, the novelty and diversity of the generated sequences assume equal importance. Moreover, sequences warranting costly wet-lab scrutiny are typically those exhibiting pronounced divergence, since revalidating the activity of already-documented peptides is scarcely justified. Although all downstream metrics ultimately quantify the degree of divergence, the evaluation criteria adopted by state-of-the-art methodologies diverge in detail and remain heterogeneous. Below, we investigate the prevailing assessment strategies.

• Clustering [200]. Clustering candidate sequences against their parental set is a widely adopted strategy to quantitatively demarcate novelty and to safeguard sequence diversity. For instance, PepDiffusion implements clustering by using CD-HIT software [256] with a threshold of 0.6.
• Percentage of peptides that are different from the real AMPs [140].
• Difference between the generated peptides and the training AMP set. For instance, for each generated sequence, Wang et al. [184] searched the training dataset for a peptide that has the smallest Levenshtein distance [202] from it and normalized the distance according to its length. Then they calculated the average length as the novelty.
• Similarity among the generated peptides, measured by calculating the Levenshtein distance between every two sequences and normalizing it by the sequence length [184], [198].
• Relative similarity [176], [186] of two sets of sequences through the global alignment algorithm [203].
• Language model perplexity. Das et al. proposed PepCVAE [183] and used a character-level LSTM language model (LM) to estimate the perplexity (PPL) of the generated sequences.
• Relative n-gram entropy [183] estimated by mixing generated samples with original samples at a 1:1 ratio
• Number of shared n-grams [257] for different values of n between the generated sequences and the original ones [183].
• Uniqueness [140], [184]. Uniqueness denotes the percentage of unique peptides generated.
• Physicochemical characteristics [140], [172], [198]. Physicochemical attributes of the generated candidates, such as charge and amphiphilicity, can be predicted by bioinformatic tools, e.g., modlAMP [122]. Discrepancies between the physicochemical property distributions of the generated sequences and those of extant AMPs likewise serve as indicators of novelty and diversity.

**Biological criteria.**

• Biosynthesis criteria [198], [200]. To approximate expert assessments of peptide synthesizability, it is better to implement the biosynthesis criteria. For instance, sequences are excluded if (i) any pentapeptide window contained more than three cationic residues, (ii) three or more consecutive hydrophobic residues (Ala, Val, Leu, Ile, Phe, Trp, Met) or three identical residues in succession were present, or (iii) Cys is detected.
• Virtual screening for biological phenotype [140], [182], [199]. Some wet-lab results can be predicted through in-silico filters, affording an additional layer of pre-experimental filtration. For instance, the hemolysis score is predicted by HemoPI [258], and the toxicity score is predicted by ToxinPred [259], [260].

## Appendix B AMP feature extraction strategies

We surveyed the feature-extraction strategies that underpin current AI-driven AMP design and adopted the five core paradigms previously systematized by Notin et al. [204], as follows.

**Global descriptors**

• Amino acid composition (AAC) [261], [262], [263], [264], [265]. AAC represents the proportion of each amino acid in a peptide sequence.
• Pseudo-amino acid composition (PAAC) [261], [262], [263], [265], [266]. The feature set of PAAC comprises over 20 attributes, incorporating various physicochemical properties such as hydrophobicity values, hydrophilicity, side chain masses, and sequence-order information [267].
• Composition, transition and distribution descriptor (CTDD) [261], [262], [263], [264], [265], [268]. These features consist of 13 different physicochemical properties, including hydrophobicity, normalized van der Waals volume, polarity, polarizability, charge, secondary structures, and solvent accessibility, which can be extracted by a Python package *iFeature* [269].
• Grouped amino acid composition (GAAC) [261], [262]. The GAAC divides the 20 AAs into five groups, which consist of the aliphatic group, aromatic group, positive charge group, negative charge group, and uncharged group.
• Dipeptide composition (DPC) [262], [264], [265], [266]. DPC aims to retrieve information about AAs using regional amino acid ordering by counting how frequently any two adjacent residues appear. Thus, the feature dimension is 400 [270].
• Tripeptide composition (TPC) [262]. Similar to DPC but for three adjacent residues [271].
• K-mer [268]. K-mer represents the occurrence frequency of all peptide sub-sequence with a length k.
• Distance-Pairs (DP) [268]. It is effective to represent the composition and position information by introducing a concept called “distance-pair”.
• Distance-based Top-n-gram approach (DT) [268]. It integrates the evolutionary information and the sequence information into a single vector.
• Composition of k-spaced amino acid pairs (CKSAAP) [262], [265]. The CKSAAP proposed to extract protein structure-related features [272].
• Conjoint triad (CT) [262]. Conjoint triad considered the properties of one AA and its vicinal AAs and regarded any three continuous AAs as a unit [273].
• ProtDCal [274], [275]. ProtDCal is a software that can provide 45,494 features, based on features from individual residues such as hydrophobicity and electronic charge [276].

**Sequence-based representations**

• Sequence (one-hot) [200], [277], [278], [279]: Each of the 20 AAs is initially encoded as a unique one-hot vector. A zero-padding approach is employed to ensure that all peptide sequences are aligned to a uniform length. As a result, a sequence S of length L turns into an L×20 matrix.
• Sequence (tokenization) [111], [170], [280], [281], [282], [283], [284]: The sequences comprise AAs represented by official IUPAC single-letter symbols.^5^ Additionally, two special class tokens are inserted at the beginning and end of the sequence to denote it as a sentence, respectively. In cases where the sequence length is less than the fixed maximum length, padding ([PAD]) tokens are used to fill the empty spaces, ensuring that all sequences have a consistent feature dimension. These standardized sequences are then embedded into X-dimensional vectors to serve as input features.
• BLOSUM62 [278], [279]. The BLOSUM62 matrix [285] aims to assess the similarity of protein sequences, which is a widely employed method for indicating protein evolutionary information. To encode a given peptide sequence, each residue is represented as a vector possessing 20 dimensions. As a result, a sequence S of length L turns into an L×20 matrix.
• Z-Scale [278]. The Z-Scale matrix [286] aims to represent amino acid physicochemical properties by characterizing each amino acid with five numerical values, including hydrophobicity and hydrophilicity, steric bulk properties and polarizability, polarity, and electronic effects. Therefore, a peptide S of length L can be encoded as an L×5 matrix.
• AAIndex [261], [264], [279]. AAindex is a comprehensive database that encompasses 531 physicochemical properties. Each of the 20 AAs is assigned a specific numerical value corresponding to each property within the AAindex database [287].
• Position-specific scoring matrix (PSSM) [266]. PSSM [288] is employed in biological sequences to express patterns, represented in a two-dimensional matrix, where the number of rows is determined by the length of the peptide sequence, while the number of columns is determined by the size of the alphabet (20).

**Graph-based representations**

• The graph-based representations are better for capturing geometry-related features, consisting of nodes and edges. Nodes can be sequences, atoms, or residues, and edges can be chemical bonds, ingredient attribution, or the geometric distance between the nodes. For instance, LABAMPsGCN [137] is a graph convolutional neural network. Its nodes consist of k-mer residues and sequences, and its edges are determined by affiliation relationships.

**Three-dimensional representations**

• Three-dimensional coordinates [206]. The three-dimensional coordinates provide information about the distances between amino acid residues, reflecting their spatial interactions. The positions of the $C_{\alpha }$ atoms in all standard AAs are selected to represent the residue-level coordinates.

**Data-driven representations**

• Embeddings (LLM or pLM) [209], [282]: By utilizing a Large Language Model (LLM) or pre-trained protein Language Model (pLM), e.g., BERT or ESM2, the embedding features for each peptide sequence extracted from the language model can be transferred to any task requiring a numerical representation.
• Predicted protein secondary structure (PPSS) [274]. Protein secondary structure refers to the local conformation of the polypeptide backbone of proteins. For instance, the secondary structure feature of all peptides predicted from DeepCNF [319] has eight types, including 3 types for helix, 2 types for strand, and 3 types for coil.
• Predicted surface-level features [206]. The geometric and chemical features for each vertex on the protein surface can be extracted from the three-dimensional structures of peptide sequences predicted by AlphaFold2 [72]. Each vertex is characterized by five geometric or physicochemical properties [320].
• Cellular automata image (CAI) [144]. By generating image representations for biological sequences, the cellular automata image approach could find some implicit sequence features [321], [322].

## Appendix C Databases and models

Public AMP databases are summarized in Table C.1, whereas the snapshot of AI methods for designing AMPs is presented in Table C.2.

## Footnotes

1 Negative charge in minority cases. For instance, *dermcidin* is negatively charged, but its activities can be enhanced by combination with zinc or highly cationic residues [108], [109], [110].

## Footnotes

2 A model is composed of multiple modules. For example, a convolutional neural network comprises convolutional modules, pooling modules, and fully connected modules, etc.

3 Liu et al. [155] proposed TransSAFP, a deep learning-guided model for the de novo design of antimicrobial materials, which integrates non-natural amino acids for enhanced peptide self-assembly and effectively predicts the functional activity of the self-assembling peptide materials.

4 Prior to the wet experiments, the biological filtering criteria were also conducted, which is used to approximate expert evaluations of peptide synthesizability [198].
